# Effects of a Modern Virtual Reality 3D Head-Mounted Display Exergame on Simulator Sickness and Immersion Under Specific Conditions in Young Women and Men: Experimental Study

**DOI:** 10.2196/41234

**Published:** 2022-11-29

**Authors:** Julia Ciążyńska, Michał Janowski, Janusz Maciaszek

**Affiliations:** 1 Department of Physical Activity and Health Promotion Science Poznan University of Physical Education Poznan Poland; 2 Department of Athletics, Strength and Conditioning Poznan University of Physical Education Poznan Poland

**Keywords:** virtual reality, HMD, simulator sickness, immersion, physical activity, exergame, Zephyr, gender differences, WHO recommendation, young adult, digital health, energy expenditure, exercise game

## Abstract

**Background:**

Many young adults do not reach the World Health Organization’s minimum recommendations for the amount of weekly physical activity. The virtual reality 3D head-mounted display (VR 3D HMD) exergame is a technology that is more immersive than a typical exercise session. Our study considers gender differences in the experience of using VR games for increasing physical activity.

**Objective:**

The aim of this study was to examine the differences in the effects of VR 3D HMD gaming in terms of immersion, simulator sickness, heart rate, breathing rate, and energy expenditure during two 30-minute sessions of playing an exergame of increasing intensity on males and females.

**Methods:**

To examine the effects of the VR 3D HMD exergame, we experimented with 45 participants (23 males and 22 females) exercising with VR 3D HMD Oculus Quest 1, hand controllers, and Zephyr BioHarness 3.0. Players exercised according to the Audio Trip exergame. We evaluated the immersion levels and monitored the average heart rate, maximum heart rate, average breathing rate, maximum breathing rate, and energy expenditure in addition to simulator sickness during two 30-minute exergame sessions of increasing intensity.

**Results:**

Audio Trip was well-tolerated, as there were no dropouts due to simulator sickness. Significant differences between genders were observed in the simulator sickness questionnaire for nausea (*F*_2,86_=0.80; *P*=.046), oculomotor disorders (*F*_2,86_=2.37; *P*=.010), disorientation (*F*_2,86_=0.92; *P*=.040), and total of all these symptoms (*F*_2,86_=3.33; *P*=.04). The measurements after the first 30-minute VR 3D HMD exergame session for all the participants showed no significant change compared to the measurements before the first 30-minute exergame session according to the total score. There were no gender differences in the immersion (*F*_1,43_=0.02; *P*=.90), but the measurements after the second 30-minute exergame session showed an increase in the average points for immersion in women and men. The increase in the level of immersion in the female group was higher than that in the male group. A significant difference between genders was observed in the average breathing rate (*F*_2,86_=1.44; *P*=.04), maximum breathing rate (*F*_2,86_=1.15; *P*=.047), and energy expenditure (*F*_2,86_=10.51; *P*=.001) measurements. No gender differences were observed in the average heart rate and maximum heart rate measurements in the two 30-minute sessions.

**Conclusions:**

Our 30-minute VR 3D HMD exergame session does not cause simulator sickness and is a very immersive type of exercise for men and women users. This exergame allows reaching the minimum recommendations for the amount of weekly physical activity for adults. The second exergame session resulted in simulator sickness in both groups, more noticeably in women, as reflected in the responses in the simulator sickness questionnaire. The gender differences observed in the breathing rates and energy expenditure measurements can be helpful when programming VR exergame intensity in future research.

## Introduction

### Background

Several studies have shown evidence that excessive sedentary behavior can harm the health of people [1-4] and that programmed physical activity may increase health-related fitness [5,6]. According to the new recommendations of the World Health Organization, adults (18-64 years) should exercise for 150-300 minutes a week at moderate intensity or 75-150 minutes at vigorous intensity [7]. For moderate intensity activities, it is about 22-42 minutes of physical activity a day to improve health. Adults should also engage in a muscle-strengthening exercise of moderate or high intensity that engages all the major muscle groups for 2 or more days per week [7]. However, it should be emphasized that this is an absolute minimum that is often not achieved [8,9]. Studies have shown that young female adults typically engage lesser in physical activity than men [10], but both genders have difficulty achieving the World Health Organization recommendations for weekly physical activity [11]. Globally, in 2016, more than a quarter of all adults were not performing enough physical activity [11]. The world of computer devices is often blamed for low activity and a sedentary lifestyle [12], and the group at risk is people in their 20s and 30s because they are the most active gamers [13]. Over the past 2 decades, the number of female video game players has increased, and females today make up half of the gaming population [14]. The enthusiasm of young people should be channelized to play virtual reality (VR) games because of the positive enhanced heart rates and energy expenditure during exergames compared to those while playing on traditional computer displays [15]. In 2016, the VR games market began to develop dynamically, and the equipment used for VR gaming was modernized. Most studies until 2016 showed that women users experienced high simulator sickness and low immersion while using the same VR play equipment and content as men [16-20]. Males and females may be sensitive to different features of the simulated environment and may experience simulator sickness accordingly (females more for 2D environments, while males for 3D environments). Further, different studies have reported increased simulator sickness differences between men and women according to the type of the utilized head-mounted display (HMD) equipment and categories of VR content (female users experienced more discomfort compared to males when exposed to high emotional and arousing content). Recent studies have shown that proper modern equipment and properly selected game content can minimize the feelings of simulator sickness and increase immersion in both genders [21-24]. However, the previous studies do not refer to exergames based on programmed physical exercises in the VR 3D HMD. No study has shown VR exergaming as a form of physical activity that can be performed for fulfilling the weekly physical activity recommendations of the World Health Organization for adults.

Immersive VR 3D HMD exergaming systems are plain body movements and are close to the planned, structured, and repetitive elements of a typical moderate training session [25]. Immersion is one of the main factors required for enabling the game users to perceive all aspects to create a real-life impression [16]. VR 3D HMD exergame is a technology that is more immersive than a typical exercise session [20]. An exergame that is a whole-body exercise is important, and it should involve rhythmic activity from large muscle masses, require quick shuffling to change positions, and should include locomotor tasks or even defensive actions to promote increased energy expenditure [26].

### Goal of This Study

Our study presents a VR device as a tool to reach the minimum recommendations of the World Health Organization for the amount of weekly physical activity and to counteract a sedentary lifestyle. As per the World Health Organization recommendation, we divided the exergame sessions into two 30-minute sessions to assess whether the minimum recommendation could be achieved by both the genders. The 30-minute sessions have also been used in another HMD study [27] and has shown a positive response in the mental health of young adults. We chose individuals in the age group of 19-29 years as the most vulnerable to a sedentary lifestyle. The aim of this study was to evaluate the effects of a modern 3D HMD exergame on simulator sickness and immersion under specific conditions in healthy people. Thus, the risk of influence of other factors such as poor health, chronic diseases, and low endurance was reduced. In our experiment, we chose such conditions for playing the exergame to minimize gender differences, and we matched the game type for a group of recipients. We chose Oculus Quest 1 with Audio Trip, a first-person perspective game with 3D vision and a low emotional VR environment, for both genders. As the sessions increased in intensity, we collected data on the heart rate, breathing rate, and energy expenditure.

### Hypotheses

We formed 3 hypotheses in this study.

1. Hypothesis 1: playing Audio Trip with a modern VR 3D HMD would result in higher cardiovascular function in men.
2. Hypothesis 2: playing Audio Trip with a modern VR 3D HMD would not cause simulator sickness in both genders.
3. Hypothesis 3: playing Audio Trip with a modern VR 3D HMD would have an immersion level that is gender independent, regardless of the playing time.

## Methods

### Participants

Sample size estimations were conducted using G*Power 3.1 (Axel Buchner). Sample size estimations were done for all analyses, and the final targeted sample size was selected such that the analysis requiring the largest number of participants can be adequately powered. Based on meta-analyses, we expect a medium effect size of Cohen *f*=.26. The sample size required to reach a level of significance of .05 with a power of .80 is 26 participants. By making 3 measurements considering gender and an assumed dropout rate of 25%, we needed to recruit 33 people to test our hypotheses. We evaluated all the participants who signed up; there was a total of 45 participants. All the participants completed this study. To be eligible for participation in this study, the individual had to be a physical education student, be between 19 and 29 years old, and have a good health condition (no neurological disorders, disability, mental disorders, psychotropic medicines, injuries, or fresh injuries), have a score of 10 or less on the Ruffier Squat Test scale, and have had no interactions with VR equipment or similar VR stimulation before. Individuals were invited to participate in the study through personal invitations and emails.

### Technology

The VR Oculus Quest 64 GB system (Oculus Quest system software, Facebook Inc, released on May 21, 2019) was used as the immersive VR technology in this study. It consists of a wireless headset through which the VR environment can be viewed and played with 2 hand controllers that enable interaction with the VR environment. People wearing glasses could also take part in this study because a special overlay for glasses was applied. To ensure proper performance, the room size should be at least 2 meters × 2 meters. The VR Oculus Quest 64 GB has a display of 5.7 inches, resolution of 2880 pixels × 1600 pixels, reference resolution of 1440 pixels × 1600 pixels, organic light emitting diode display type, and refresh frequency of 72 Hz. The name of the game that was used was Audio Trip and was distributed from the Oculus Quest store app.

### Instruments

Four methods were used for the usability evaluation: Audio Trip VR exergame, Simulator Sickness Questionnaire (SSQ), The Immersion Questionnaire, and BioHarness 3.0 measurements. One of the inclusion criteria for the study was the Ruffier Squat Test.

**Figure 1:**
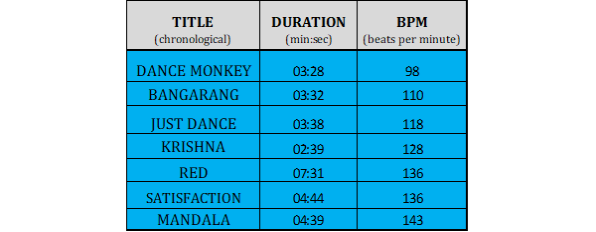
Exergame playlist in beginner and regular modes of the Audio Trip virtual reality game, including the title, duration of songs, and beats per minute. BPM: beats per minute.

The purpose of the Audio Trip VR game (Andromeda Entertainment) [28] is to dance to the rhythm and immerse into the virtual musical and fitness environment. A player is obliged to follow the colored path by using their hands to catch 2 colored gems, smash drums, and dodge virtual barriers. In this study, players had to follow a special 30-minute playlist 2 times in the beginner and regular modes. A playlist is a proprietary idea in which the beats per minute increase during play, and the difficulty level increases in the second session, thereby increasing the intensity. The details of the playlist are shown in Figure 1.

The SSQ is one of the most frequently used simulator testing tools [29]. In this research, we used the SSQ by Kennedy et al [30], translated into Polish by Biernacki et al [31]. One of the essential aspects of research on simulators is how different test conditions affect the severity of the symptoms due to a simulator. Among the conditions in this study, one should specify both the movement of the platform and the type of visual stimuli. This questionnaire evaluates 26 symptoms due to the simulator. Questions on nausea (question numbers 1, 8, 10, 11, 12, 22, 25), oculomotor disorders (question numbers 1, 2, 5, 6, 7, 12, 15), and disorientation (question numbers 7, 1, 14, 15, 16, 17, 18) were asked in the questionnaire. The task of the person examined using SSQ consists of making a subjective assessment of the severity of specific symptoms. A 4-step scale was used: (1) none, no symptoms due to the simulator; (2) small, few symptoms due to the simulator; (3) moderate, moderate symptoms due to the simulator; and (4) significant, serious symptoms due to the simulator. The SSQ result includes both the overall levels of the symptoms from using a simulator (SSQ total), and the indicators consist of individual (unportioned relative to each other) scales: (1) nausea symptoms of increased salivation, sweating, nauseousness, and burping; (2) oculomotor disorder symptoms of fatigue, headache, eye fatigue, and difficulty in concentration; and (3) disorientation symptoms of dizziness, daze feeling (both with open and closed eyes), and blurriness (out of focus). The first step when calculating SSQ is to convert the results to a numerical form. The SSQ scales are expressed on a 4-stage Likert scale (where 0=none, 1=small, 2=moderate, and 3=significant). For calculating the score for SSQ, add raw values for the data of the symptoms assigned to the specified factor and multiply by a specific number. For individual scales, they are as follows: (1) nausea, 9.54 (score ranging from 0 to 200.34); (2) oculomotor disorders, 7.58 (scores ranging from 0 to 159.18); (3) disorientation, 13.92 (scores ranging from 0 to 292.32); and (4) SSQ total, 3.74 (scores ranging from 0 to 235.62).

The Immersion Questionnaire is a scale measuring video game engagement [32]. In this research, we used the Immersion Questionnaire by Jennett et al [32], which was translated into Polish by Strojny et al [33]. This questionnaire was used as a tool for measuring the player’s absorption/immersion while playing games. Researchers commonly consider immersion to be an important part of the videogame user experience [34-37]. The Polish adaptation consists of 27 test items instead of 31 questions of the original questionnaire, because 4 questions in the original questionnaire had a low correlation with the overall test score and are unnecessary [38]. The Immersion Questionnaire scales are expressed on a 5-stage Likert scale (where 1=very small, 2=small, 3=average, 4=more than average, 5=a lot/definitely). The total score to earn on the Immersion Questionnaire is 135.

A portable wireless piezoelectric recording system (BioHarness 3.0, OmniSense 3.9.7, Zephyr Technology Corp) [39] was used for monitoring energy expenditure, estimated according to the following formula: energy expenditure (kcal) = gender × (–55.0969 + 0.6309 heart rate + 0.1988 weight + 0.2017 age) + (1 – gender) × (–20.4022 + 0.4472 heart rate – 0.1263 weight + 0.074 age) (gender: 1 for male, 0 for female; heart rate, including average heart rate and maximum heart rate; and breathing rate, including average breathing rate and maximum breathing rate).

In the Ruffier Squat Test, participants are made to do 30 squats in 45 seconds [40]. Heart rate is recorded before the test (P1), at the end of 45 seconds (P2), and at 1 minute after the test (P3). The test score is calculated in the form of an index—the Ruffier Index expressed as (P1 + P2 + P3) – 200/10. The range of the Ruffier Index given was 0 to 17, but higher than 10 is an adaptation to insufficient effort or even poor adaptation. The individual ranges are as follows: 0 is a very good adaptation to effort, between 0 and 5 is a good adaptation to effort, and between 5 and 10 (including 10) is the ability to adapt to the average effort. Ruffier originally developed this test for testing the European population.

### Procedure

**Figure 2:**
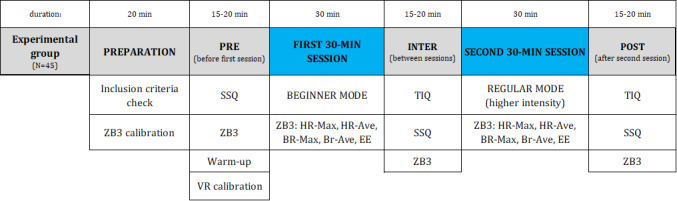
Study protocol presented in the chronological order from left to right for the experimental group. Br-Ave: average breathing rate; Br-Max: maximum breathing rate; EE: energy expenditure; HR-ave: average heart rate; HR-Max: maximum heart rate; SSQ: Simulator Sickness Questionnaire; TIQ: The Immersion Questionnaire; VR: virtual reality; ZB3: Zephyr Bioharness 3.0.

Each participant came once to be examined and approved for research and to take part in the main study. Each participant attended an information meeting. They were informed of the study and encouraged to ask questions, after which they signed a written consent form if they agreed to participate. The study protocol is presented in a chronological order in Figure 2. The participants filled in the first 2 pages of SSQ (see Multimedia Appendix 1). The first page included preliminary information before starting the study about simulator experience and physical statements, that is, level of current physical fitness, the occurrence of diseases during a week, alcohol consumption, medications taken 24 hours before the study, and sleep duration. The second page collected information on cybersickness data before the first 30-minute VR 3D HMD exergame session. Before completing the SSQ, a BioHarness 3.0 sensor was attached to the chest by a strap and calibrated with a computer. The participants were weighed and their heights were measured, and then these data were entered into the OmniSense software (Zephyr BioHarness 3.0).

**Figure 3:**
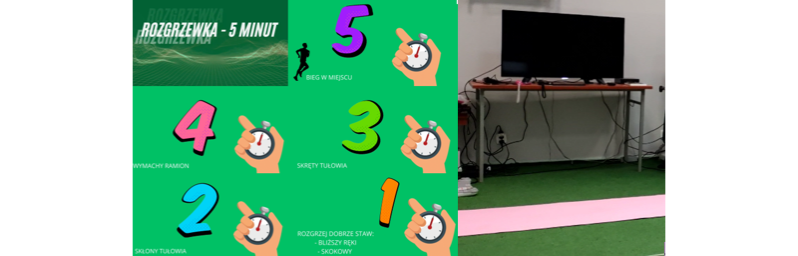
The various elements of the 5-minute warm-up (5: running in place, 4: arm swings, 3: torso twists, 2: torso bends, 1: warming the joints, that is, wrist and ankle) on the television.

**Figure 4:**
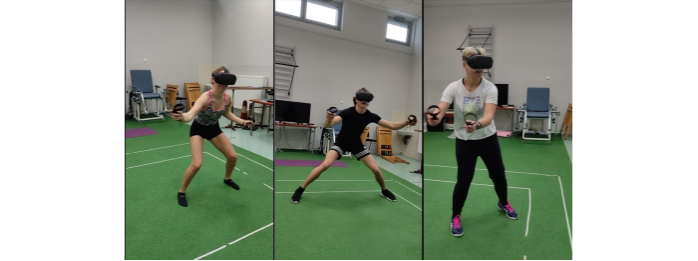
Virtual reality exergame intervention in a safe area of 2.5 meters × 2.5 meters.

**Figure 5:**
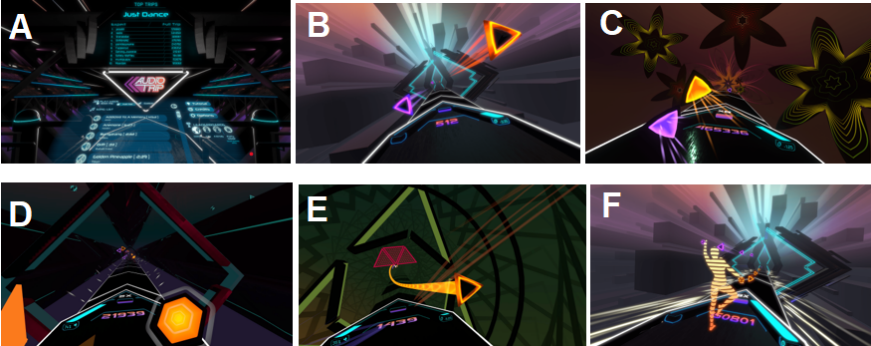
View of the Audio Trip game. From left: A. Selecting a track from the list.; To the rhythm of the music, hitting two-colored triangles as intended: B. touching by R/L hand C. hitting at a certain angle by R/L hand, D. smashing the drums by R/L hand, E. following the path by R/L hand and dodging the barriers.; F. Following the trainer (back perspective).

Each participant took part in a 5-minute warm-up, according to the instructions in a video prepared earlier (see Figure 3). The VR 3D exergame tutorial starts with the calibration of HMD and controllers. People wearing glasses could also take part in this study because a special overlay for glasses was applied. Moreover, left-handed people could take part in this study. The participants stood next to an imaginary console with songs and were then fitted with the VR equipment. At this point, researchers had a preview of the VR world on the screen of the mobile phone to be able to see what the participant saw during the experimental sessions. The safe space for the participant to move was a square of 2.5 meters × 2.5 meters. In the VR environment, the controllers were represented by hands in the console environment and by 2-color orbs in the exergame environment. The tutorial was built in steps, with each step being supported by instructions on what tasks to perform to meet the goal to proceed to the next step, and the tasks were graded from the easiest to the most difficult (see Multimedia Appendix 2). The details of the playlist are shown in Figure 1. The intensity of the game increases during each session and with the difficulty level. The participants were dressed in casual sports clothes (Figure 4) and had safety face pads between the HMD Oculus Quest headset and their face. The details on what the Audio Trip game looks like are shown in Figure 5. We determined whether a participant managed to perform each task by noting the points for every 7 songs from a playlist on the checklist box. Between the songs was a quick pause to select the next one from the playlist.

After the first 30 minutes of the exergame playlist session, participants proceeded to the measurements that were taken between the 2 sessions: The Immersion Questionnaire, SSQ, and BioHarness 3.0 measurements (Figure 2). The participants were expected to answer the questions alone, but some questions required clarification and affirmation by the researcher to confirm the proper interpretation. After completing the measurements in the interval between the 2 sessions, the second session of the exergame started. The interval time of 15-20 minutes was enough for the heart rates of all the participants to return to normal resting heart rate before the start of the next game session. The game was then launched on the regular level. After completing the second 30-minute playlist (60 minutes in total), the same measurements were performed as done between the 2 sessions.

### Data Interpretation

We analyzed the data measured from BioHarness 3.0 in the experiment by using the OmniSense software. The data measured from the experiment are maximum heart rate, average heart rate, maximum breathing rate, average breathing rate, and energy expenditure. Repeated-measures analysis of variance (ANOVA) was used to test the hypotheses. We analyzed the data using Statistica 13.3 (TIBCO Software Inc), and statistical significance was defined as *P*≤.05.

### Ethics Approval

This study was performed according to the ethical standards laid down in the Declaration of Helsinki. All participants provided signed informed consent, and the Bioethics Commission of Poznan University of Medical Sciences granted the ethical approval for this study (decision 32/20).

## Results

### Participant Characteristics

The mean age (in years) of the participants (N=45) was 21.69 (SD 2.76; range 19-28). The mean body weight (in kilograms) was 71.03 (SD 13.07; range 44.5-106.5). The mean body height (in centimeters) was 173.98 (SD 8.09; range 155.6-186.1). The mean body mass index (in kg/m²) was 23.3 (SD 3.23; range 18.4-32.3). The experimental group was mixed; females constituted 49% (22/45) of the study population (Table 1).

**Table 1:** Characteristics of the study population (N=45).

Participant characteristic	Value
Mean age (years)	21.69
Mean body weight (kg)	71.03
Mean body height (cm)	173.98
Mean BMI (kg/m²)	23.3
Females (n)	22
Ruffier index	6.5

### SSQ Results

#### Nausea

Significant differences between genders were observed in nausea symptoms (Table 2), that is, increased salivation, sweating, nauseousness, and burping. Analysis of the results with the ANOVA test showed that the size of the changes in the mean values for the SSQ nausea score was *F*_2,86_=0.80 (*P*=.46).

**Table 2:** Gender differences in nausea during the 3 measurements.^a^

Simulator sickness questionnaire on nausea		Female group	Male group
**Before the first 30-minute exergame session**			
	Average (SD)	9.54 (9.74)	10.37 (9.22)
	Minimum points	0	0
	Maximum points	28.62	38.16
**Between the exergame sessions**			
	Average (SD)	13.01 (10.54)	18.25 (11.19)
	Minimum points	0	0
	Maximum points	38.16	47.7
**After the second** **30-minute exergame session**			
	Average (SD)	20.38 (13.05)	22.40 (12.87)
	Minimum points	0	0
	Maximum points	57.24	47.7

^a^*F*_2,86_=0.80; *P*=.046.

#### Oculomotor Disorders

Significant differences between genders were observed in oculomotor disorder symptoms (Table 3), that is, fatigue, headache, eye fatigue, and difficulty in concentrating. Analysis of the results with the ANOVA test showed that the size of the changes in the mean values for the SSQ oculomotor disorders score was *F*_2,86_=2.37 (*P*=.10).

**Table 3:** Gender differences in the simulator sickness questionnaire for oculomotor disorders during the 3 measurements.^a^

Simulator sickness questionnaire on oculomotor disorders			Female group		Male group
**Before the first 30-minute exergame session**					
	Average (SD)	18.26 (14.18)		14.50 (12.25)	
	Minimum points	0		0	
	Maximum points	60.64		30.32	
**Between the exergame sessions**					
	Average (SD)	22.74 (13.32)		11.21 (14.01)	
	Minimum points	0		0	
	Maximum points	53.06		30.32	
**After the second** **30-minute exergame session**					
	Average (SD)	34.11 (18.80)		21.09 (15.33)	
	Minimum points	0		0	
	Maximum points	83.38		53.06	

^a^*F*_2,86_=2.37; *P*=.010

#### Disorientation

Significant differences between genders were observed in disorientation symptoms (Table 4), that is, dizziness, daze feeling (both with open and closed eyes), and blurriness (out of focus). Analysis of the results with the ANOVA test showed that the size of the changes in the mean values for the SSQ disorientation score was *F*_2,86_=0.92 (*P*=.40).

**Table 4:** Gender differences in the simulator sickness questionnaire on disorientation during the 3 measurements.^a^

Simulator sickness questionnaire on disorientation		Female group	Male group
**Before the first 30-minute exergame session**			
	Average (SD)	11.39 (11.42)	7.87 (10.05)
	Minimum points	0	0
	Maximum points	41.76	27.84
**Between the exergame sessions**			
	Average (SD)	16.45 (15.67)	5.45 (7.49)
	Minimum points	0	0
	Maximum points	55.68	55.68
**After the second 30-minute exergame session**			
	Average (SD)	24.68 (22.80)	15.13 (10.65)
	Minimum points	0	0
	Maximum points	69.60	83.52

^a^*F*_2,86_=0.92; *P*=.004.

#### SSQ Total Symptoms

**Figure 6:**
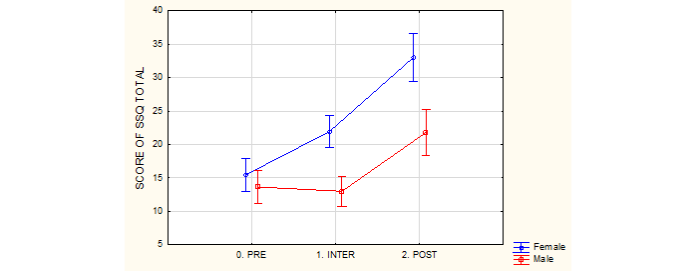
Gender differences in the total simulator sickness score during the three measurements. PRE: before the first 30-minute exergame session; INTER: between the exergame sessions; POST: after the second 30-minute exergame session; SSQ: Simulator Sickness Questionnaire.

Audio Trip was well-tolerated, as there were no dropouts due to simulator sickness. Significant differences between genders were observed in SSQ total symptoms (oculomotor disorders + nausea + disorientation). Analysis of the results with the ANOVA test showed the size of changes in mean values for the SSQ total symptoms was *F*_2,86_=3.33 (*P*=.04); however, after the first 30-minute session, all participants showed no significant change compared to premeasurements according to SSQ total symptoms results (Table 5 and Figure 6). One-way ANOVA showed no significant differences between the measurements for men (*P*=.83) and women (*P*=.59) before the first 30-minute exergame session and between the sessions.

The two 30-minute sessions of the exergame changed the SSQ profile only in the male group. The SSQ profile of males changed during the 3 measurements as follows (highest score to the smallest score): oculomotor disorders>nausea>disorientation before the first session, nausea>oculomotor disorders>disorientation between the 2 sessions, and nausea>oculomotor disorders>disorientation after the second session. However, the SSQ profile of the female group remained the same in every measurement, that is, before, between, and after the exergame sessions, as follows: oculomotor disorders>disorientation>nausea. The mean points for the SSQ profiles are described in Table 6.

**Table 5:** Gender differences in the simulator sickness questionnaire for the total symptoms during the 3 measurements.^a^

Simulator sickness questionnaire on the total symptoms			Female group		Male group
**Before the first 30-minute exergame session**					
	Average (SD)	15.47 (11.41)		13.65 (10.68)	
	Minimum points	0		0	
	Maximum points	48.62		33.66	
**Between the exergame sessions**					
	Average (SD)	21.93 (11.55)		13.01 (9.22)	
	Minimum points	0		0	
	Maximum points	52.36		33.66	
**After the second 30-minute exergame session**					
	Average (SD)	32.98 (17.30)		15.13 (16.60)	
	Minimum points	0		0	
	Maximum points	74.80		59.84	

^a^*F*_2,86_=3.33; *P*=.04.

**Table 6:** Gender differences for the simulator sickness questionnaire profile during the 3 measurements.

Simulator sickness questionnaire profile			Female group		Male group
**Before the first 30-minute exergame session (mean points)**					
	Oculomotor disorder	18.26		14.50	
	Nausea	9.54		10.37	
	Disorientation	11.39		7.87	
**Between the exergame sessions (mean points)**					
	Oculomotor disorder	22.74		11.21	
	Nausea	13.01		18.25	
	Disorientation	16.45		5.45	
**After the second** **30-minute exergame session (mean points)**					
	Oculomotor disorder	34.11		21.09	
	Nausea	20.38		22.40	
	Disorientation	24.68		15.13	

### The Immersion Questionnaire

**Figure 7:**
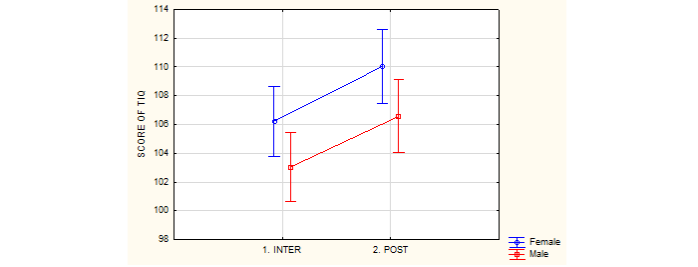
Gender differences in the immersion questionnaire score during the two measurements. INTER: between the exergame sessions; POST: after the second 30-minute exergame session; TIQ: The Immersion Questionnaire.

No significant differences between genders were observed in the Immersion Questionnaire score (Table 7 and Figure 7). Analysis of the results with the ANOVA test shows the size of changes in the mean values for the Immersion Questionnaire score (*F*_1,43_=0.02; *P*=.89).

**Table 7:** Gender differences in the immersion questionnaire during the 2 measurements.^a^

Immersion questionnaire			Female group		Male group
**Between the exergame sessions**					
	Average (SD)	106.23 (7.80)		103.04 (10.68)	
	Minimum points	72		78	
	Maximum points	121		121	
**After the second** **30-minute exergame session**					
	Average (SD)	110.05 (11.06)		106.56 (10.83)	
	Minimum points	79		76	
	Maximum points	129		129	

^a^*F*_1,43_=0.02; *P*=.89

### BioHarness Measurements

A significant gender difference was observed in the average breathing rate (*F*_2,86_=1.44; *P*=.04), maximum breathing rate (*F*_2,86_=1.15; *P*=.047), and energy expenditure (*F*_2,86_=10.513; *P*=.001) measurements (Table 8). No gender differences appeared in the average heart rate and maximum heart rate measurements in the two 30-minute sessions.

**Table 8:** Gender differences in the average heart rate, maximum heart rate, average breathing rate, maximum breathing rate, and energy expenditure during the 3 measurements.

BioHarness measurements			Female group		Male group		Interaction, *F* test *(df)*			*P* value	
**Average heart rate**								0.19 (2,86)			.83
	Before the first 30-minute exergame session	90.95		80.87							
	Between the exergame sessions	142.72		129.86							
	After the second 30-minute exergame session	144.68		133.56							
**Maximum heart rate**								0.29 (2,86)			.75
	Before the first 30-minute exergame session	110.77		101.26							
	Between the exergame sessions	171.82		158.17							
	After the second 30-minute exergame session	172.77		160.52							
**Average breathing rate**								1.44 (2,86)			.04
	Before the first 30-minute exergame session	14.50		12.91							
	Between the exergame sessions	27.95		28.99							
	After the second 30-minute exergame session	27.05		28.70							
**Maximum breathing rate**								1.15 (2,86)			.047
	Before the first 30-minute exergame session	23.09		21.65							
	Between the exergame sessions	37.27		35.04							
	After the second 30-minute exergame session	36.27		36.65							
**Energy expenditure**								10.513 (2,86)			.001
	Before the first 30-minute exergame session	33.82		39.47							
	Between the exergame sessions	267.95		340.96							
	After the second 30-minute exergame session	272.77		356.82							

## Discussion

### Principal Findings

#### Hypothesis 1: Audio Trip Exergame With Modern VR 3D HMD Would Result in Higher Cardiovascular Function in Men

The findings from this study provide support for our first hypothesis. A significant gender difference was observed in the average breathing rate (*F*_2,86_=1.44; *P*=.04), maximum breathing rate (*F*_2,86_=1.15; *P*=.047), and energy expenditure (*F*_2,86_=10.513; *P*=.001) measurements. Seebauer et al [41] suggested that the effect of exercise intensity on the occurrence of coordination between breathing rate and rhythms of exercise differs between men and women. A similar mechanism could have occurred in our study due to the use of a rhythmic music game. During heavy exercise, tidal volume plateaus and increase in minute ventilation can only be reached via an increase in the breathing rates and energy expenditure. Interestingly, even though women have smaller lung volumes compared to men [42], in our study, men adjusted their breathing rates more during the whole exergame session. Trinschek et al [43] proved that men showed significantly higher values in cardiorespiratory variables (by 12%-34%) during the running treadmill test until exhaustion [43]. Men users feel more excited when they do something they like very much or want to achieve something and are forced to breathe more frequently during an exercise session [44]. Additionally, the frequency of breathing increases energy expenditure [45]. Breathing can also change in response to changes in emotions. Due to the physical activity, the attractiveness of the task, perceived competence, and autonomy concerning emotional experience are especially important aspects [46]. Although our study showed a prominent level of immersion, it can be assumed that satisfaction influenced the need to increase breathing in male users. The values of average heart rate and maximum heart rate showed no gender differences in both sessions because the groups were well-matched in terms of physical performance, as per the Ruffier Squat Test inclusion criterion in this study.

#### Hypothesis 2: Audio Trip Exergame With Modern VR 3D HMD Will Not Cause Simulator Sickness in Both Genders

The findings from our study provide partial support for our second hypothesis. There were statistical differences between the genders in both sessions: men were more resistant to simulator sickness than women. After two 30-minute sessions, there was a change in simulator sickness (*F*_2,86_=3.33; *P*=.04); however, after the first 30-minute session, all participants showed no significant change compared to premeasurements according to SSQ total results. Although there is a lack of studies about gender imbalance in the susceptibility to simulator sickness in the context of modern HMDs, some studies do elucidate this point [23,24,47]. Female participants may experience discomfort only in specific conditions (movements in first-person perspective). Males and females may be sensitive to different features of the simulated environment and experience simulator sickness accordingly (females more for 2D environments, while males for 3D). Studies have shown the causes for simulator sickness in men and women, which are different by the type of the utilized HMD equipment and categories of VR content (female participants experienced more discomfort compared to males when exposed to highly emotional and arousing content) [22]. A systematic review on gender differences in VR HMD [23] showed that women are commonly more sensitive to simulator sickness when the realism of the content is low or when the game is highly emotional. Such an increase in SSQ total score in women can be partially explained that female users may be more willing to report distress and discomfort during the experiment [48]. This may be due to the social biological differences and social expectations [49]. The newest HMD technology can provide a more advantageous experience [27,50] and if this is combined with a careful selection of VR content, the risk of VR simulator sickness can be leveled off. Furthermore, Giammarco et al [51] suggested that gender differences in SSQ scores may be differentially related to gender differences in cognitive functions like spatial attention depending on the VR environment. Another aspect is that the VR HMD simulator sickness has been found to increase after 10 minutes [52]. Our study showed that only the second session (a total of 60 minutes of VR exergaming) of VR content caused statistically significant differences in simulator sickness. Therefore, the hypothesis was confirmed partially, because only the first 30-minute of the Audio Trip exergame will not cause simulator sickness in adults, which is a positive finding in the field of SSQ. A new type of VR HMD and appropriately selected content and intensity can help delay the appearance of the effects of simulator sickness for men and women, as shown in other VR studies [50,53]. Szpak et al [53] suggested that the exposure duration did not influence any visual measure, and observable changes in accommodation and vergence did occur within the first 10 minutes of VR exposure and did not significantly change for exposures up to 50 minutes. The results of our study should encourage men and women to try VR HMD exercise, without fearing that playing may have a high probability of causing them discomfort during 30 minutes of exergaming. The development of VR equipment and more modern games in the future should extend the effect of non–simulator sickness.

#### Hypothesis 3: Immersion Level is Gender Independent Regardless of the Playing Time

The findings from this study provide support for our third hypothesis. There were no gender differences in the level of immersion (*F*_1,43_=0.02; *P*=.90) but the second 30-minute session showed an increase in the average points for both women and men. The increase in the level of immersion in the female group was higher than that in the male group. As we mentioned, immersion is one of the main factors required to enable the game users to perceive all aspects to create a real-life impression [16]. Studies until 2011 show that men are more frequent immersive users of VR devices. Men, regardless of their prior game experience, expressed more sensory immersion and more control over the environment than women. The golden period of VR was the year 2016. Since 2016, VR devices were contemporary and were found to be suitable for users (weightless, wireless, etc). Our study suggests that there are no gender differences concerning the level of immersion with contemporary VR equipment (Oculus Quest) if the VR content is suitable for both genders. Our experiment was performed with physical education students who had not interacted with VR equipment or similar VR stimulation before, and we made sure that the content of the game was interesting for both genders (physical exercise for physical education students). Physical exergame content (Audio Trip) and technical capabilities (wireless HMD, hand controllers) can probably contribute to the reduction of gender differences.

### Limitations

The first limitation in this study was the assessment of the breathing rate based on chest movements alone. Based upon the principle of a strain gauge sensor, thoracic expansion and contraction cause size differentials that induce changes in capacitance because of the resultant changes in impedance. The change in impedance is manifested as a change in the waveform signal amplitude represented as a sine wave with downward and upward deflections, indicating chest expansion (increased impedance) and contraction (decreased impedance), respectively [54]. Incorrect tension on the chest strap may prevent adequate sensor response to chest expansion and contraction [39]. To eliminate the effects of these variances, we recommend the use of additional tools such as spirometry for future studies measuring physiological effort and energy expenditure.

The second limitation in this study was the acceptance of the necessity to be a physical education student as one of the inclusion criteria in this study. The aim was to study the effects the VR 3D HMD exergame on fully healthy and fit people. This allowed reducing the risk of the influence of other factors, for example, poor health, chronic diseases, and low endurance. In addition, what we tried to highlight in our study, which mattered the most, was the interest in the type of game and the gathering of the appropriate number of people for research who would meet the inclusion criteria, including the Ruffier Squat Test score. We chose people interested in sports by the type of studies selected (physical education students).

### Conclusions

This study showed that a 30-minute session of the VR 3D HMD exergame does not cause simulator sickness and is a very immersive type of exercise for men and women users. This exergame gives the opportunity to reach the minimum recommendations for the amount of weekly physical activity for adults. The second exergame session resulted in simulator sickness in both groups, more noticeably in women, as indicated by the responses in the SSQ. Gender differences were observed in the breathing rate and energy expenditure measurements, and this information can be helpful when programming VR exergame intensities in future research. Our findings provide a foundation for future research on VR exergames with no simulator sickness but as a refreshing and an enjoyable type of exercise for male and female users. Motivating the public to be physically active is perhaps one of the most important and difficult tasks. Gamification of physical exercises can facilitate more weekly physical activity than that recommended by the World Health Organization for minimum weekly physical activity for adults. Future immersive exergaming concepts should focus on increasing the intensity while considering user susceptibility to simulator sickness.

## Appendix

Multimedia Appendix 1 The simulator sickness questionnaire.

Multimedia Appendix 2 Audio Trip virtual reality tutorial video.

## Abbreviations

ANOVA: analysis of variance
HMD: head-mounted display
SSQ: Simulator Sickness Questionnaire
VR: virtual reality
