# Supplementary material for: Effects of a Modern Virtual Reality 3D Head-Mounted Display Exergame on Simulator Sickness and Immersion Under Specific Conditions in Young Women and Men: Experimental Study
Source: JMIR Serious Games. 2022 Nov 29;10(4):e41234. doi: 10.2196/41234 (PMC9748792; doi:10.2196/41234)
Supplement: Multimedia Appendix 1 [file games_v10i4e41234_app1.docx]

**Imię i nazwisko:** ...................................................................................................................................................................

**Data badania:** ...................................................................................................................................................................

**Informacje wstępne przed przystąpieniem do badania**

1. Ile czasu minęło od Twojego ostatniego badania na symulatorze? ................................ dni
2. Ile czasu minęło od Twojego ostatniego lotu samolotem? ................................ dni
3. Ile czasu minęło od Twojej ostatniej morskiej podróży? ................................ dni
4. Ile czasu minęło od ostatniego razu, kiedy wykonywałeś/aś zadanie w warunkach wirtualnej rzeczywistości? ................................ dni
5. Jakie masz inne doświadczenie w przebywaniu na urządzeniach/sprzęcie wywołujących wrażenie nietypowego ruchu? ......................................................................................................................................................................................................................................................................

**Stan fizyczny przed przystąpieniem do badania (kwestionariusz stanu fizycznego)**

1. Czy obecnie Twoja sprawność fizyczna jest na typowym dla Ciebie poziomie? (zaznacz kółkiem): tak nie

Jeżeli zaznaczyłeś/aś „nie”, podaj przyczynę: ..........................................................................................................................................................

1. Czy w ostatnim tygodniu chorowałeś/aś? (zaznacz kółkiem)

Tak nie

Jeśli zaznaczyłeś/aś „tak”, podaj: rodzaj choroby (przeziębienie, grypa itp.): ...................................................................................................................................................................................

a) nasilenie choroby (zaznacz na osi): bardzo łagodne bardzo poważne

b) czas trwania choroby: ................. godz. / dni

c) główne objawy choroby/dolegliwości: ............................................................................................................................................................

d) czy obecnie czujesz się w pełni zdrowy/a?

tak nie

1. Ile poniżej wymienionych porcji alkoholu spożyłeś/aś w ciągu ostatnich 24 godz.?

................. 330 ml piwa ............... 100 ml wina .......... 30 ml mocnego alkoholu, np. wódki

4. Wskaż, zaznaczając kółkiem, wszystkie lekarstwa, jakie zażywałeś/aś w ciągu ostatnich 24 godz.:

a) żadne b) środki nasenne lub uspokajające c) aspiryna, tylenol, inne przeciwbólowe d) leki przeciwhistaminowe e) leki zmniejszające przekrwienie f) inne (proszę wymienić): ...................................................................................................................................................................5. Ile godzin spałeś/aś ostatniej nocy? ........................... godz.

6. Czy ta ilość snu była wystarczająca? (zaznacz kółkiem) tak nie

7. Podaj wszelkie inne uwagi dotyczące Twojego obecnego stanu fizycznego, które mogą wpływać na wydajność podczas przeprowadzanego badania: ...................................................................................................................................................................

**Poziom wyjściowy (Pre)**

Instrukcja: Wypełnij poniższy kwestionariusz, ZANIM rozpocznie się badanie na symulatorze. Zaznacz KÓŁKIEM odpowiedzi opisujące, w jakim stopniu odczuwasz w tej chwili wymienione niżej symptomy.

* Polegające na iluzji ruchu, najczęściej wirowego (otoczenia lub osoby, która go doświadcza).
** Wzrokowe wrażenie ruchu podczas przebywania poza symulatorem, samochodem czy samolotem.
*** Dolegliwości żołądkowe dotyczą dyskomfortu przypominającego krótkotrwałe nudności.
 **STOP!!! Osoba przeprowadzająca badanie powie, kiedy je kontynuować.**

**Poziom po badaniu 30-minutowym (INTER)**

Instrukcja: Wypełnij poniższy kwestionariusz, ZANIM rozpocznie się badanie na symulatorze. Zaznacz KÓŁKIEM odpowiedzi opisujące, w jakim stopniu odczuwasz w tej chwili wymienione niżej symptomy.

* Polegające na iluzji ruchu, najczęściej wirowego (otoczenia lub osoby, która go doświadcza).
** Wzrokowe wrażenie ruchu podczas przebywania poza symulatorem, samochodem czy samolotem.
*** Dolegliwości żołądkowe dotyczą dyskomfortu przypominającego krótkotrwałe nudności.
**INFORMACJE PO BADANIU (INTER)**

1. Czy kiedy znajdowałeś/aś się w symulatorze, doświadczałeś/aś wrażenia ruchu (np. czy miałeś/aś poczucie własnego ruchu)? Zaznacz kółkiem jedną odpowiedź: tak nie trochę

2. W skali od 1 (słabo) do 10 (doskonale) oceń poziom wykonania przez siebie zadania na symulatorze: ..........................................

3. Czy w trakcie badania doświadczyłeś/aś czegoś dziwnego?

Zaznacz kółkiem jedną odpowiedź: tak nie.
Jeśli zaznaczyłeś/aś „tak”, opisz to odczucie:..................................................................................... ......................................................................................................................................................................................................................................................................................................................................

**Poziom po badaniu 60-minutowym (POST)**

Instrukcja: Wypełnij poniższy kwestionariusz, ZANIM rozpocznie się badanie na symulatorze. Zaznacz KÓŁKIEM odpowiedzi opisujące, w jakim stopniu odczuwasz w tej chwili wymienione niżej symptomy.

* Polegające na iluzji ruchu, najczęściej wirowego (otoczenia lub osoby, która go doświadcza).
** Wzrokowe wrażenie ruchu podczas przebywania poza symulatorem, samochodem czy samolotem.
*** Dolegliwości żołądkowe dotyczą dyskomfortu przypominającego krótkotrwałe nudności.
**INFORMACJE PO BADANIU (POST)**

1. Czy kiedy znajdowałeś/aś się w symulatorze, doświadczałeś/aś wrażenia ruchu (np. czy miałeś/aś poczucie własnego ruchu)? Zaznacz kółkiem jedną odpowiedź: tak nie trochę

2. W skali od 1 (słabo) do 10 (doskonale) oceń poziom wykonania przez siebie zadania na symulatorze: ..........................................

3. Czy w trakcie badania doświadczyłeś/aś czegoś dziwnego?

Zaznacz kółkiem jedną odpowiedź: tak nie.
Jeśli zaznaczyłeś/aś „tak”, opisz to odczucie:..................................................................................... ......................................................................................................................................................................................................................................................................................................................................
